# Left Distal Transradial Approach for Coronary Intervention: Insights from Early Clinical Experience and Future Directions

**DOI:** 10.1155/2019/8671306

**Published:** 2019-10-31

**Authors:** Hao Feng, Zhenfei Fang, Shenghua Zhou, Xinqun Hu

**Affiliations:** Department of Cardiology, The Second Xiangya Hospital of Central South University, Changsha, China

## Abstract

Left distal transradial approach is a novel technique for coronary intervention. This technique is convenient for specialists to operate and welcomed for right-handed patients. The anatomical snuffbox and the first intermetacarpal are two available puncture sites on the basis of hand anatomy. In technical aspects, main differences between left distal transradial approach and conventional transradial approach are patient's special position, puncture procedure, sheath choice, and hemostasis methods. According to the preliminary data, this technique is feasible and safe and it has low rate of complications including radial artery occlusion in forearm. Left distal transradial approach is a quite promising strategy of coronary intervention and deserves further exploration. In this review article, we describe the main technical characteristics and the results obtained from early clinical experiences. We also discuss the main challenges and future perspectives on this novel technique.

## 1. Introduction

Transradial approach (TRA) was first introduced for percutaneous coronary intervention (PCI) by Kiemeneij in 1993 [1]. Since then, TRA has gained a huge increasing popularity among interventional specialists and patients undergoing percutaneous coronary and peripheral diagnostic and revascularization procedures [2]. Compared to transfemoral approach, TRA offers several outstanding advantages, such as less vascular complications, less hospital stays, and more mobilisation [3]. Now, TRA is considered as a default strategy for PCI [4]. TRA also has a few drawbacks. One of the most prevalent complications is radial artery occlusion (RAO) which occurs in 2.8%–11.7% of patients despite proper anticoagulation [5]. Because of the dual blood supply of forearm, generally RAO is asymptomatic and ignored, though sometimes paresthesia and distal ischemic could happen [6]. Notably, this segment of radial artery is greatly useful in coronary artery bypass grafting, hemodialysis fistula preparation, and repeated PCI [5, 7]. Therefore, it is necessary to prevent the occurrence of RAO.

During the transradial procedures, right radial approach is generally preferred in routine clinical practice mainly due to the convenience of manipulation for the operators from patient's right side and the current design of radial compression devices for the right wrist in medical market [8, 9]. However, under some circumstances, the operator needs to cross over to the left radial artery. Main reasons are right RAO, sclerosis, extreme tortuosity, and nondevelopment right radial artery [10]. Additionally, puncture in left radial artery would be welcomed by majority of patients who are right-handed and would no longer endure the restriction of right hand after PCI [11]. In conventional left transradial approach, the left arm is in volar position and the operator has to bend over the patient, which leads to the situation where the operator will be exposed to higher radiation doses. In order to solve the problem, Kiemeneij [10] proposed a novel left distal transradial approach (ldTRA) in anatomical snuffbox in 2017. The objectives of this review were to provide an overview of the main technical characteristics and to summarize the early clinical results. We also discuss the main challenges experienced in the initial phases of clinical application and future perspectives on this novel technique.

## 2. Anatomy of Hand Circulation and Alternative Puncture Sites

The radial artery descends along the lateral side of forearm, and it is palpable between the tendon of the flexor carpi radialis medially and the anterior border of the radius [12], where the conventional TRA are operated.

**Figure 1:**
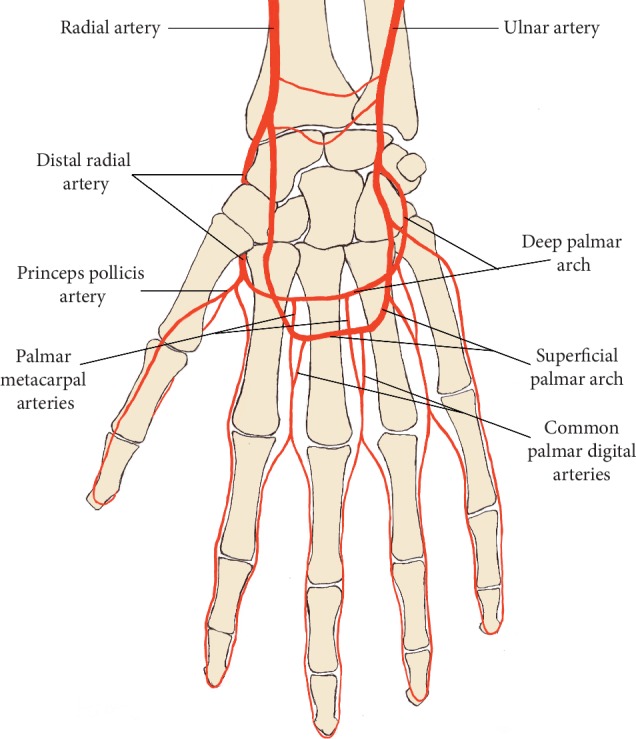
Anatomy of the distal forearm and hand artery circulation (from palmar side).

At the wrist, the radial artery firstly gives rise to the superficial palmar branch, which passes through the thenar muscles, anastomosing with the end of the ulnar artery to form the superficial palmar arch. Distally, the radial artery curls posterolaterally to pass on the dorsal aspect of the carpus under the tendon of dorsal muscles, and then it goes under the second metacarpal bone to the palm side and connects with the deep branch of ulnar artery to complete deep palm arch. Blood supply to the digits is mainly supplied by the interconnected palmar metacarpal arteries and common palmar digital arteries arising from the deep palmar arch and the superficial palmar arch, respectively [12–14] (Figure 1).

There are 2 sites where the pulse of radial artery in the dorsum of hand can be felt. They are, respectively, the anatomical snuffbox and the first intermetacarpal space, which were proposed as the puncture site of distal radial artery recently [15].

**Figure 2:**
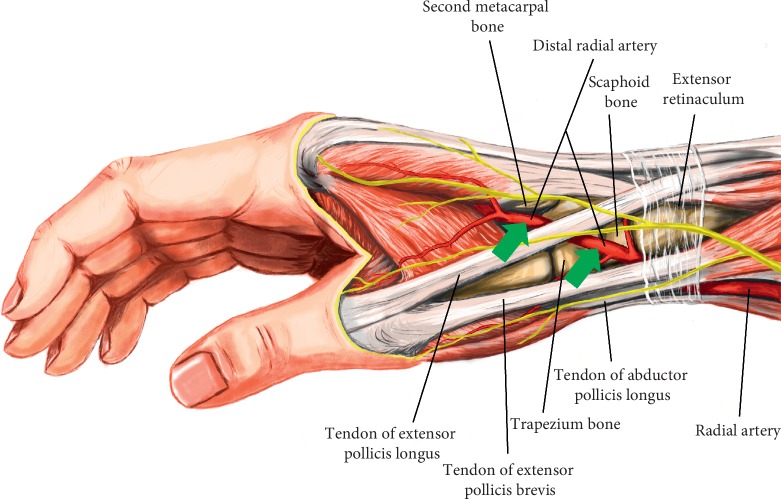
Puncture sites of distal radial artery (green arrows) and relevant surrounding anatomic structures.

The anatomical snuffbox (radial fossa and fovea radialis) is a triangular depression space on the radial, dorsal aspect of the hand, showing up when the thumb is extended [16]. It is surrounded laterally by the tendons of abductor pollicis longus and extensor pollicis brevis muscles, medially by the tendon of extensor pollicis longus muscle, and posteriorly by extensor retinaculum of wrist [10–15]. The anatomical snuffbox has a “bone basement” composed of scaphoid bone and the trapezium bone and a ceiling of thin soft tissue under the skin [15]. Consequently, radial artery in this area is easily palpable and compressed to hemostasis (Figure 2).

Another available puncture site of distal radial artery is the first intermetacarpal space, precisely in the vertex of the angle between the long extensor and the second metacarpal bone [15]. As a continuity of the radial artery in the anatomical snuffbox, the radial artery in this area is superficial too (Figure 2).

## 3. Technical Aspects

The main differences between ldTRA and conventional TRA are patient's special position, puncture procedure, sheath choice, and hemostasis methods. After the sheath introduced, the intervention operation of ldTRA is similar with TRA.

### 3.1. Patient's Preparation

All the researches highlight the presence of valid pulse in the distal puncture site to confirm the well-development of distal radial artery. Some operators suggest that the ultrasound should be applied to detect the diameters, bifurcation, and depth of the artery [17, 18].

The patient's left hand is asked to bent over towards the right groin, and the operator takes a position near the patient's head. To bring the artery to the surface of the fossa, the patient is asked to grasp his thumb under the other four fingers or hold a roll of gauze, with the hand slightly abducted [10].

### 3.2. Puncture Procedures

**Figure 3:**
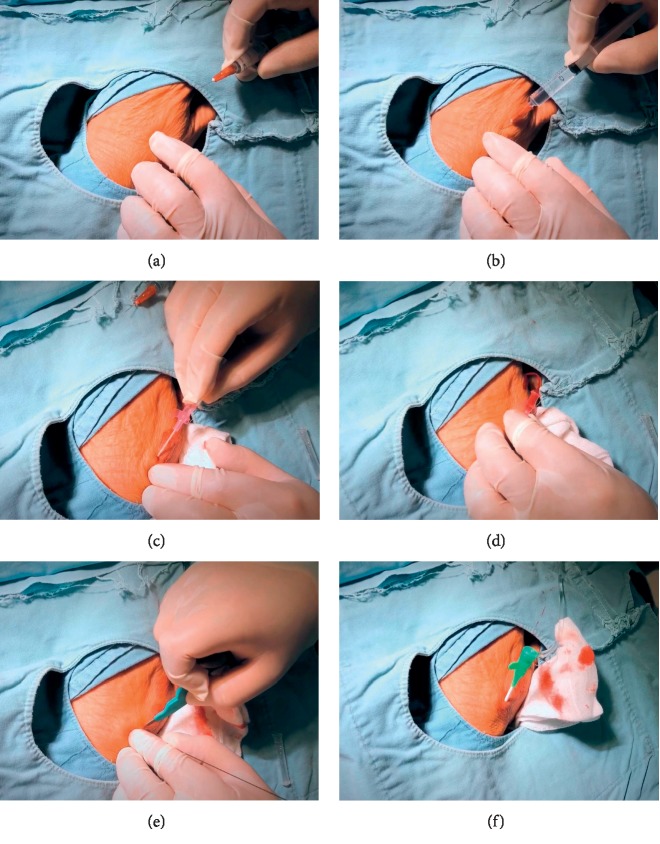
Puncture procedure in the first intermetacarpal space. (a) Confirmation of the most powerful pulse site. (b) Local anesthesia by using lidocaine. (c) Puncture angle is less than 30 degree from lateral to media. (d) Successful puncture. (e) A small skin incision is made before introducing the sheath. (f) 6 Fr sheath is in situ.

After disinfection and local anesthesia (Figure 3(b)), the artery is punctured according to operator's experience by using a micropuncture needle or a cannula-over-needle (Figure 3(c)). 20G or 21G needle is recommended [10, 19]. The angle of puncture is varied. Kiemeneij [10] suggested 30–45 degree from lateral to media, while Lee et al. [17] claimed that the angle should be less than 30 degree to avoid the periosteal pain. In order to avoid puncturing into one of the terminal branches, the puncture is performed at the proximal part of the anatomical snuffbox or the first intermetacarpal space (Figure 3(a)).

After successful artery puncture, a guidewire was smoothly advanced through the needle and used to guide the sheath through the artery (Figures 3(d), and 3(f)). There is insufficient information about distal radial artery, although its diameter is generally considered to be smaller. Accordingly, using a small-diameter introducer sheath seems to be a wise choice [15]. As previously reported, the 6 Fr sheath is mostly used [10, 18–21]. However, recently Gasparini et al. [22] demonstrated that ldTRA using a 7 Fr sheath for coronary chronic total occlusion (CTO) interventions is feasible and safe. Thus, if necessary, operators can still use larger sheath without the need to change the vascular access. Operators can make the choice according to the experience and ultrasound.

Notably, the site of distal radial artery puncture is about 5 cm distally to the classic wrist-level site, so extra-length catheters need to be prepared. This point was also stressed by K. Shingo in *TCT2018* (*Transcatheter Cardiovascular Therapeutics 2018*). For the purpose of preventing damage to the tip of the introducer and sheath, which might damage the artery, a small skin incision is made [10] (Figure 3(e)).

### 3.3. Hemostasis

After the procedure, hemostasis is obtained. In general, the methods of hemostasis that have been reported could be divided into 3 types.

The first one is to use the TR-band. According to Karim's operation [18], a larger sized radial band is used due to the larger girth of the hand at level of the anatomical snuffbox. Another similar device is a band with air bladder (*SafeGuard Radial™ Compression Device*), as proposed by Kiemneij [10], which does not need to make the whole hand oppressed. These bands are inflated of some air when the sheath is pull out and then removed in 2–3 h following the usual radial band removal protocol. Finally, the arteriotomy site is then covered with a small gauze covered by clear dressing.

The second type of hemostasis is using the gauze and elastic bandage. Actually nearly half of the published researches are applied this method [17, 21, 23, 24]. After sheath removal, the early hemostasis is obtained by manual compression, and then the puncture site is wrapped by an elastic bandage with gauze roll for about 3 hours. In fact, just gauze and bandage and no manual compression could be effective too [20].

The third type is called two-step hemostasis (Saijo Style hemostasis), proposed by K. Shingo in *TCT2018*. Stepty and elastic bandage are applied in distal puncture site and TR-band in conventional TRA site. TR-band is deflated gradually. 2 hours later elastic bandage is removed and 4 hours later, all the items are removed.

No matter what type, hemostasis in dRA is nearly achieved in 3 hours, relatively easier than classic TRA. Recently, a prospective research showed ldTRA facilitates earlier discharge of postcoronary angiography [25]. The operator can make the choice according to the preference and situation. In addition, the movement of wrist will not be restricted, so the patient will be more comfortable.

## 4. Early Clinical Experiences

Left distal transradial artery (ldTRA) approach is a novel technique originally introduced in 2017, so the outcome data so far are limited. The main clinical results of this technique are summarized in Tables 1 and 2.

Kiemeneij [10] primarily reported a series of 118 patients; among them, 70 patients (59%) underwent left distal TRA. Puncture was not attempted in other 48 patients, due to weak or absent pulse (23%), logistical reasons (6%), presence of an indwelling venous cannula (5%), left-handedness (3.5%), and patient preference (3.5%). Hemostasis was obtained within 3 h in all patients. Notably, postprocedure ultrasound assessment revealed 0% forearm radial artery occlusion, while one patient got distal radial artery occlusion. In this research, left distal TRA was unsuccessful in 11% of cases, and 2 patients had complications potentially related to the approach site: ecchymosis of the hand (*n* = 1) and minor forearm bleeding (*n* = 1). On average, the score of visual assessment scale (VAS) is low. Kiemeneij [10] also reported an unpublished total number of 656 patients with a very low rate of complications undergoing distal radial artery approach at another center.

Lee et al. [17] reported ldTRA in 187 patients, and the procedural success rates of the coronary angiography (CAG) and PCI are 100% and 92.9%, respectively. According to the results, minor hematoma occurred in 14 (7.4%) patients, and there was no distal radial artery occlusion, perforation, pseudoaneurysm, or arteriovenous fistula. Valsecchi et al. [20] reported 90% success in a straightforward series of 52 patients undergoing distal radial artery approach (79% right side approach). Failure causes were distal radial artery occlusion, puncture-mediated vasospasm, and hypoplastic snuffbox artery.

Another early experience with left distal transradial approach via anatomical snuffbox from was described by Kim et al. [21]. CAG was performed in all 132 patients who underwent successful leſt snuffbox approach. Among 42 patients who needed to perform PCI, 1 patient changed into the right femoral approach due to a severe angulated calcified lesion in the left circumflex artery. Regarding vascular complication, forearm swelling with bruising occurred in 2 (4.9%) PCI cases. Another series of 54 cases via left and right anatomical snuffbox was described by Soydan and Akın [19]. 2 patients needed to cross over to the femoral artery due to the tortuosity of radial artery. There was no occurrence of radial artery occlusion, hematoma, or hand numbness. Time of complete hemostasis was within 3 hours. All procedures were very well tolerated according to visual assessment scale, and mean hospital stay was 3 days.

More recently, Gasparini et al. [22] demonstrated that ldTRA using a 7 sheath for CTO PCIs is feasible and associated with a high procedural success rate and low vascular access-site complication rates. In this research, technical success and procedural success were, respectively, achieved in 70.3% and 78.1% of 41 patients. No bleeding and spasm happen after procedure, and 4.3% of patients developed dRAO.

Compared to the conventional TRA, Koutouzis et al. [24] demonstrated in a randomized trial that ldTRA is associated with lower successful cannulation rates, prolonged duration of cannulation, and increased number of attempts and number of skin punctures. However, this did not affect the total procedural time, which was similar between ldTRA and TRA. He supposed increased tortuosity and angulations at the distal puncture site would be the reason of high failure rate.

## 5. Main Challenges and Future Directions

On the basis of the shared data, the high success rate and low complication rate of the distal radial artery approach, especially the ldTRA, suggest that this novel technique is safe and feasible. Substantially, ldTRA has a few important advantages over conventional TRA approach. First of all, right-handed patients have no longer to be bothered by the restrained movement of right hand after catheterization. During the procedures, patients are asked to put the left hand on the abdomen and near the right groin with the thumb under the other four fingers, which is a relatively natural and comfortable position for patients. Besides, this position will enable doctors operate on the right side rather than bend over the patient, which is quite cumbersome. Therefore, the doctor could work at a safe distance from the radiation source. Another important advantage is less hemostasis time. According to recent data, the hemostasis of most patients could be obtained in 3 hours [25]. The reason is probably that the distal radial artery lies superficial in anatomical snuffbox and 1^st^ intermetacarpal space. In addition, the reported average VAS score is low, meaning that patients are well tolerated of the pain in ldTRA.

All the studies demonstrate very low complication rates, including the rate of RAO. It is known that RAO is the most common complication in TRA [6]. The main causes of RAO are the injury of intima of radial artery and local blood flow interruption, resulting in the formation of thrombosis at the puncture site [26, 27]. However, the puncture site of ldTRA is distal from the wrist and smaller sheath (mostly 6 Fr) is selected, so the intimal injury in the conventional TRA site is slight. Sgueglia et al. [15] reported that distal blood flow was slower when radial artery was compression in the wrist than in the distal site. These two factors may be the reason of low rate of RAO in ldTRA. Interestingly, recently it is reported that the ldTRA could recanalize the proximal radial artery total occlusion; hence, ldTRA seems not only to obviate the RAO but also to solve RAO [28].

The main drawback is the challenging puncture of a small and weak artery, with a steeper learning curve. This is the important reason of puncture failure. All the relative researches emphasized the necessity of valid pulse in the puncture site of ldTRA. Some also apply ultrasound to affirm the good condition of distal radial artery. Kim et al. [29] reported the average diameter of radial artery in anatomical snuffbox was 2.57 mm in 101 Korean individuals, while 2.65 mm at the wrist. He pointed the woman has a smaller diameter and higher failure puncture rate of distal radial artery than the man. Thus, maybe men are more suitable for ldTRA than women.

On the other hand, the smaller diameter of distal radial artery could make the use of larger size of sheath and guider catheter impossible. This can affect the success of complex procedures such as CTO PCI by using ldTRA. Plus, the choice of guide catheter internal diameter may limit the support for crossing complex lesions and may preclude the use of particular techniques, such as IVUS and microcatheters. However, a recent work shows that ldTRA using 7 Fr sheath is feasible and safe [22]. Therefore, if necessary, ldTRA could still be an alternative strategy to dealing with complex procedures like CTO PCI.

Another problem is the length of catheters. Most catheters are designed for conventional puncture site at present, so these devices could be not long enough when the puncture site is about 5 cm blow the conventional site. Consequently, doctor needs to operate in the terminal of catheters [20].

Indeed, ldTRA can cause distal radial artery occlusion (dRAO) correspondingly [1]. A distinctive feature of this technique is a puncture site proximal from the pollicis brevis artery and distal from the branch supplying the superficial palmar arch [30]. Thus, an occlusion at this site will not affect the antegrade blood flow to superficial palmar arch, and there is the retrograde flow from ulnar artery in deep palmar arch. Digits blood flows are maintained, preventing ischemia and hand disability. Notably, sometimes the superficial and deep palmar arch are incomplete or undeveloped, which could increase the risk of hand ischemia in case of RAO or dRAO [15, 31].

At present, most researches have applied the left distal approach at the anatomical snuffbox. The attempts in the first metacarpal space are rare, which is possibly because of the bigger difficulty and higher failure rate. However, this opinion needs more studies to support.

Although the ldTRA seems more ideal than the conventional TRA, preliminary data are very limited. Outcome comparison versus conventional site at the wrist level is especially insufficient. Large sum and multicenter series are eagerly awaited to complete the procedure protocols, including indications, sheath choice, length of devices, and best hemostasis type and finally to determine whether this new technique could be the default strategy or just an alternative choice of conventional TRA.

## Figures and Tables

**Table 1:** Patient data from preliminary researches of distal transradial approach.

Author	Year	Cases	CAG	PCI	Reasons				
					STEMI	NSTEMI	UAP	SAP	Others
Kiemeneij F	2017	70	43 (61)	25 (36)	6 (9)	17 (24)	6 (9)	28 (40)	15 (21)
Lee JW	2018	200	187 (98)	87 (47)	17 (9)	45 (23)	74 (37)	38 (19)	26 (13)
Valsecchi O	2018	52	52 (100)	0 (0)	NA	NA	NA	34 (66)	13 (25)
Kim Y	2018	150	132 (88)	42 (48)	2 (1)	NA	NA	NA	NA
Soydan E	2018	54	54 (100)	20 (37)	10 (19)	6 (11)	1 (2)	NA	NA
Gasparini GL	2019	41	0 (0)	41 (100)	0 (0)	0 (0)	0 (0)	0 (0)	41 (100)

CAG: coronary angiography; PCI: percutaneous coronary intervention; SAP: stable angina pectoris; STEMI: ST-segment elevation myocardial infarction; NA: not available; NSTEMI: non-ST elevation myocardial infarction; UAP: unstable angina pectoris.

**Table 2:** Procedural data from preliminary researches of distal transradial approach.

Author	Puncture success	Procedural success^*∗*^	Sheath			PT (min)	FT (min)	Major complications		
			5 Fr	6 Fr	7 Fr			Hematoma	RAO	dRAO
Kiemeneij	66 (94)	62 (89)	22 (31)	40 (58)	0 (0)	24.8	9.6	1 (1.5)	0 (0)	1 (1.5)
Lee JW	191 (96)	190 (95)	41 (25)	62 (33)	1 (1)	35.6	11.3	14 (7.4)	0 (0)	0 (0)
Valsecchi	47 (90)	47 (90)	1 (2)	50 (96)	0 (0)	43	NA	NA	NA	NA
Kim	140 (93)	132 (88)	0 (0)	132 (88)	0 (0)	NA	NA	0 (0)	0 (0)	0 (0)
Soydan	54 (100)	52 (96)	0 (0)	54 (100)	0 (0)	NA	9.6	0 (0)	0 (0)	0 (0)
Gasparini	37 (90)	32 (78)	0 (0)	5 (12.2)	32 (78.1)	NA	61.4	0 (0)	0 (0)	2 (4.3)

^*∗*^Procedural success: CAG or PCI is completed successfully by using ldTRA. dRAO: distal radial artery occlusion; Fr: French; FT: fluoroscopy time; NA: not available; PT: procedural time; RAO: radial artery occlusion.
